# Evaluation of the Physical and Oxidative Stabilities of Fish Oil-in-Water-in-Olive Oil Double Emulsions (O_1_/W/O_2_) Stabilized with Whey Protein Hydrolysate

**DOI:** 10.3390/antiox12030762

**Published:** 2023-03-21

**Authors:** Marta Padial-Domínguez, Pedro J. García-Moreno, Rubén González-Beneded, Antonio Guadix, Emilia M. Guadix

**Affiliations:** Department of Chemical Engineering, University of Granada, 18071 Granada, Spain

**Keywords:** double emulsions, omega-3 polyunsaturated fatty acids, protein hydrolysate, lipid oxidation, food enrichment

## Abstract

This work studied the physical and oxidative stabilities of fish oil-in-water-in-olive oil double emulsions (O_1_/W/O_2_), where whey protein hydrolysate was used as a hydrophilic emulsifier. A 20 wt.% fish oil-in-water emulsion, stabilized with whey protein hydrolysate (oil: protein ratio of 5:2 *w*/*w*) and with a zeta potential of ~−40 mV, only slightly increased its D_4,3_ value during storage at 8 °C for seven days (from 0.725 to 0.897 µm), although it showed severe physical destabilization when stored at 25 °C for seven days (D_4,3_ value increased from 0.706 to 9.035 µm). The oxidative stability of the 20 wt.% fish oil-in-water emulsion decreased when the storage temperature increased (25 vs. 8 °C) as indicated by peroxide and *p*-anisidine values, both in the presence or not of prooxidants (Fe^2+^). Confocal microscopy images confirmed the formation of 20 wt.% fish oil-in-water-in-olive oil (ratio 25:75 *w*/*w*) using Polyglycerol polyricinoleate (PGPR, 4 wt.%). Double emulsions were fairly physically stable for 7 days (both at 25 and 8 °C) (Turbiscan stability index, TSI < 4). Moreover, double emulsions had low peroxide (<7 meq O_2_/kg oil) and *p*-anisidine (<7) values that did not increase during storage independently of the storage temperature (8 or 25 °C) and the presence or not of prooxidants (Fe^2+^), which denotes oxidative stability.

## 1. Introduction

The food industry is interested in the fortification of food matrices with long chain omega-3 polyunsaturated fatty acids (PUFAs), such as eicosapentaenoic (EPA, C20:5n-3) and docosahexaenoic (EPA, C20:5n-3) acids, due to their positive health benefits [1]. Nevertheless, these omega-3 PUFAs are highly susceptible to oxidation during food processing and storage, reducing the nutritive and quality properties of the enriched food [2]. A common strategy to efficiently deliver omega-3 PUFAs into food matrices is the use of fish oil-in-water emulsions [3].

The fortification of water-based food matrices with fish oil-in-water emulsions has been extensively studied in the literature [4]. However, only a few studies are reported on the use of fish oil-in-water emulsions for omega-3 enrichment of lipid-based matrices, leading to the formation of oil-in-water-in-oil (O_1_/W/O_2_) emulsions. For instance, Dwyer et al. [5] investigated the use of oil (blends of camelina and fish oil)-in-water emulsions stabilized with sodium caseinate to enrich spreads with omega-3 fatty acids, indicating that a higher content of fish oil reduced the oxidative stability of the spread. The same authors [6] reported an increase in the oxidative stability of spreads enriched with oil (85% camelina:15% fish oil)-in-water emulsions by the addition of different antioxidants (e.g., water-soluble green tea extract, α-tocopherol, or a combination of both). Moreover, the spray-drying of O_1_/W/O_2_ emulsions, which was previously dispersed in an extra water phase containing encapsulating agents (e.g., lactose and sodium caseinate), has also been investigated for further protection of the inner oil due to the suitability of the resulting capsules in dry mixing [7].

Multiple emulsions (e.g., O_1_/W/O_2_) are colloidal systems with two different oil phases and two thermodynamically unstable interfaces that are kinetically stabilized by emulsifiers: (i) hydrophilic emulsifier with high hydrophile–lipophile balance (HLB) in the O_1_/W interface, and (ii) hydrophobic emulsifier with low HLB in the W/O_2_ interface [8]. Traditional hydrophilic emulsifiers used to stabilize the simple (O/W) emulsion are dairy proteins (e.g., sodium caseinate and whey protein concentrate) [5,7,9], whereas polyglycerol polyricinoleate (PGPR) is still the most effective hydrophobic emulsifier to stabilize W/O emulsions [10]. Undoubtedly, oxidative stability of O_1_/W/O_2_ emulsions is a challenge when having polyunsaturated oil phase(s). In these heterogeneous systems, the properties of the interfaces (e.g., viscoelasticity, charge, thickness), as well as the presence of antioxidants at the interface, are particularly important to reduce lipid oxidation since the interfaces are the places where lipid oxidation is initiated [11]. Therefore, the use of emulsifiers also exhibiting antioxidant properties is of special importance to assure the location of antioxidants at the interface and, thus, minimize lipid autoxidation in emulsions [12,13]. When properly designed, an enzymatic hydrolysis of proteins releases peptides with increased exposure of: (1) hydrophobic patches that allows their anchoring at the o/w interface, and (2) side chains of antioxidant amino acids [14]. Recently, whey protein hydrolysate, with a low degree of hydrolysis (10%) and which exhibits both excellent emulsifying and antioxidant (e.g., radical scavenging and metal chelating) activities, has been reported as a promising protein-derived emulsifier for the stabilization of fish oil delivery systems [15,16].

Thus, this work aimed at investigating the physical and oxidative stabilities of fish oil-in-water-in-olive oil double emulsion (O_1_/W/O_2_), which was studied as a model system for lipid-based matrices enriched with omega-3 PUFAs. The use of whey protein hydrolysate for the stabilization of the simple O_1_/W emulsion was evaluated. The physical and oxidative stabilities of the simple and double emulsions were assayed at 8 and 25 °C and in the presence or not of prooxidants (Fe^2+^). The results obtained provide new insights for the development of physically and oxidatively stable O_1_/W/O_2_ double emulsions containing fish oil as the inner oil phase.

## 2. Materials and Methods

### 2.1. Materials

Whey protein (34.6% protein content) was kindly provided by Abbott Laboratories (Granada, Spain). Alcalase 2.4 L was purchased from Novozymes (Bagsvaerd, Denmark). PGPR was kindly donated by Palsgaard A/S (Juelsminde, Denmark). Refined fish oil (Omega Oil 1812 TG Gold) was acquired from BASF Personal Care and Nutrition GmbH (Illertissen, Germany) and stored at −80 °C until use. Olive oil was purchased from a local supermarket. The fatty acid composition of the oils was determined with gas chromatography (GC) as previously described [17]. The fatty acid composition of the fish oil was as follows (major fatty acids in %, *w*/*w*): 7.0% myristic acid (C14:0), 16.7% palmitic acid (C16:0), 8.8% palmitoleic acid (C16:1n-7), 4.1% stearic acid (C18:0), 8.2% oleic acid (C18:1n-9), 19.3% eicosapentaenoic acid (C20:5n-3), and 16.1% docosahexaenoic acid (C22:6n-3). The fatty acid composition of the olive was: 14.0% palmitic acid (C16:0), 71% oleic acid (C18:1n-9), and 8.0% linoleic acid (C18:2n-6). Peroxide value (PV) of the fish and olive oils were measured as described in Section 2.6.1 and were 0.47 ± 0.06 and 5.8 ± 0.2 meq O_2_/kg oil, respectively. The oxidative stability indexes of the oils determined by Rancimat [18] were 0.40 ± 0.06 and 8.6 ± 0.2 h for fish and olive oils, respectively. The rest of reagents used for analysis were of analytical grade.

### 2.2. Enzymatic Hydrolysis

Enzymatic hydrolysis of whey protein was carried out in an automatic titrator 718 Stat Titrino (Metrohm AG, Herisau, Switzerland) to a degree of hydrolysis 10% (DH 10%) with Alcalase. For this purpose, a solution containing 36 g of protein was prepared with distilled water to a final volume of 0.9 L. The process conditions were set to 50 ºC and the pH to 8, whereas the enzyme-substrate ratio was fixed to 0.55 (*w*/*w*). The DH was estimated with the pH-stat-method, as previously described [19]. After the desired DH was reached, the enzyme was deactivated at 100 °C for 5 min. The whey protein hydrolysate (WPH) solution was freeze-dried in a Labconco freeze drying system (Kansas City, MO, USA).

### 2.3. Emulsions Preparation and Sampling

First, a simple 20% fish oil-in-water emulsion (pH 8) stabilized with WPH (oil:protein ratio of 5:2 *w*/*w*) was produced. A pre-homogenization process was carried out for 3 min at 15,000 rpm using an Ultraturrax T-25 homogenizer (IKA, Staufen, Germany) whilst the oil was added to the aqueous phase containing WPH during the first minute. The coarse simple emulsion was then homogenized in a high-pressure homogenizer (PandaPLUS 2000; GEA Niro Soavi, Lübeck, Germany) at a pressure range of 450/75 bar, applying 3 passes. Subsequently, the simple emulsion was dispersed in olive oil containing PGPR (4 wt.%) for 3 min using an Ultraturrax T-25 homogenizer (IKA, Staufen, Germany) at 18,000 rpm to obtain the double fish oil-in-water-in-olive oil emulsion. The ratio simple emulsion to olive oil was 25:75 *w*/*w*. The emulsions were stored at 8 °C and 25 °C for 6 weeks in brown bottles of 30 mL. Each bottle contained 15 g of emulsions. At both temperature values, emulsions were stored with and without addition of 100 µM FeSO_4_. Additionally, 0.05% (*w*/*w*) of sodium azide was incorporated into the primary emulsions to avoid microbial growth.

### 2.4. Microstructure of Simple O_1_/W and Double O_1_/W/O_2_ Emulsions

Double emulsions were stained with Nile Red to be observed using a confocal scanning laser microscopy instrument (Leica DMI6000 B, Germany). Oil staining was carried out by mixing 2 mL of the samples with 0.1 mL of a Nile Red solution (1 mg/mL in ethanol). WPH was stained with fluorescein isothiocyanate (0.1 mL of 1 mg/mL in ethanol). To capture the images, a 60× oil immersion objective lens was used with ×3 zoom. The images were recorded with the software Leica Microsystems, establishing the spectrums of Nile Red in 543 nm for excitation and 650 nm for emission and of fluorescein isothiocyanate in 488 nm for excitation and 520 nm for emission.

### 2.5. Physical Stability of Emulsions

#### 2.5.1. Zeta Potential

The zeta potential of the simple O_1_/W emulsion was measured at day 0 using a Zetasizer Ultra (Malvern Instruments Ltd., Worcestershire, UK) at 25 °C. Samples were previously diluted in a volume proportion of 2/1000 with distilled water. Measurements were made in triplicate.

#### 2.5.2. Droplet Size Distribution

The oil droplet size distribution of the simple O_1_/W emulsion was determined at days 0, 1, 3, and 7 using a static light scattering instrument (Mastersizer 3000, Malvern Instruments, Worcestershire, UK). Samples were diluted in recirculating water (3000 rpm) to achieve an obscuration in the range 12–15%. The refractive indexes of fish oil (1.481) and water (1.330) were used as particle and dispersant, respectively. Measurements were made in triplicate.

#### 2.5.3. Turbiscan Stability Index

Physical stability of simple O_1_/W and double O_1_/W/O_2_ emulsions was measured with a Turbiscan Lab (Formulaction, Toulouse, France) on days 0, 1, 2, 3, 6, and 7. A volume of 20 mL of each emulsion was transferred into special vials, which were then placed into the instrument and scanned measuring backscattering (BS) and transmission (T). The results obtained were used to investigate if the samples experienced creaming, sedimentation, or flocculation. The Turbiscan stability index (TSI) reported by the instrument was used for evaluation of the global physical stability.

### 2.6. Oxidative Stability of Emulsions

Samples were taken at days 0, 1, 3, and 7 and placed at −80 °C under nitrogen atmosphere until the determination of peroxide (PV) and *p*-anisidine values (AV).

#### 2.6.1. Peroxide Value (PV)

Oil extraction from simple and double emulsions for PV determination was made as follows: ca. 0.35 g of simple or 0.1 g of double emulsion were weighed and mixed with 5 mL of distilled water. Then, 20 mL of hexane/2-propanol (1:1, *v*/*v*) solvent was added and mixed for 5 min to be later centrifuged at 670× *g* for 2 min. PV was measured using the thiocyanate assay as described in Drusch et al. [20] with some modifications. Extracted oil was diluted with 2-propanol prior addition of iron-II-chloride and ammonium thyocianate solutions and then was incubated for 5 min at 25 °C. Oil extraction was made in duplicate for each sample. The PV measurements were made in triplicate for each oil extract. Results were expressed in meq O_2_ per kg of oil.

#### 2.6.2. *p*-Anisidine Value (AV)

For *p*-anisidine value determination, 1.5 g of simple or 0.4 g of double emulsion were weighed to carry out the oil extraction as described in the previous section. Oil extraction was made in duplicate for each sample. The AV measurements were made in triplicate according to the ISO 6885:2006 method [16] for each oil extract.

#### 2.6.3. Statistical Analysis

Statgraphics 18 (Statistical Graphics Corp., Rockville, MD, USA) was used for data analysis. Data were expressed as mean ± standard deviation. First, one-way ANOVA was performed to identify if there were significant differences between samples. Then, multiple sample comparison using Tukey’s test allowed us to identify means that were significantly different from each other. Differences between means were considered significant at *p* ≤ 0.05.

## 3. Results and Discussion

### 3.1. Physical Stability of Emulsions

#### 3.1.1. Physical Stability of Simple O_1_/W Emulsions

**Figure 1:**
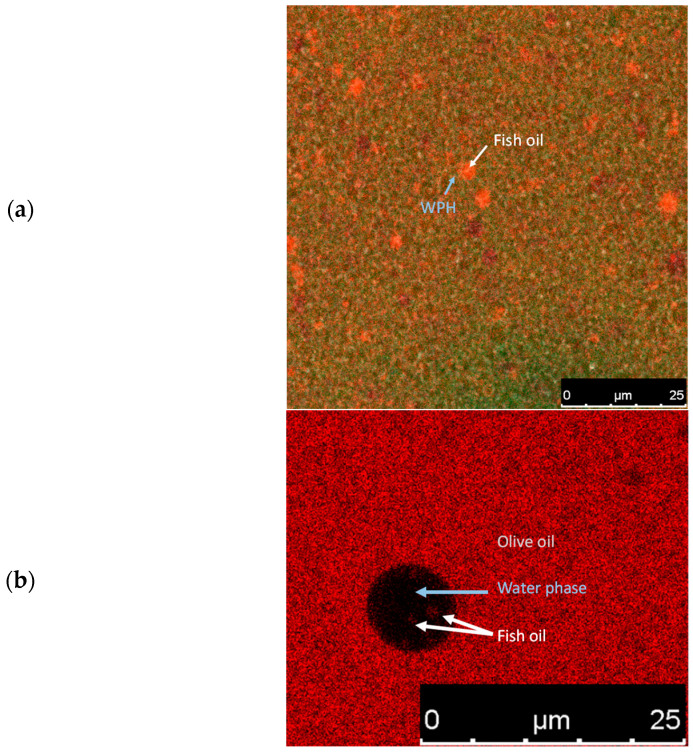
Confocal microscopy images of (**a**) simple O_1_/W emulsion and (**b**) double O_1_/W/O_2_ emulsion.

**Figure 2:**
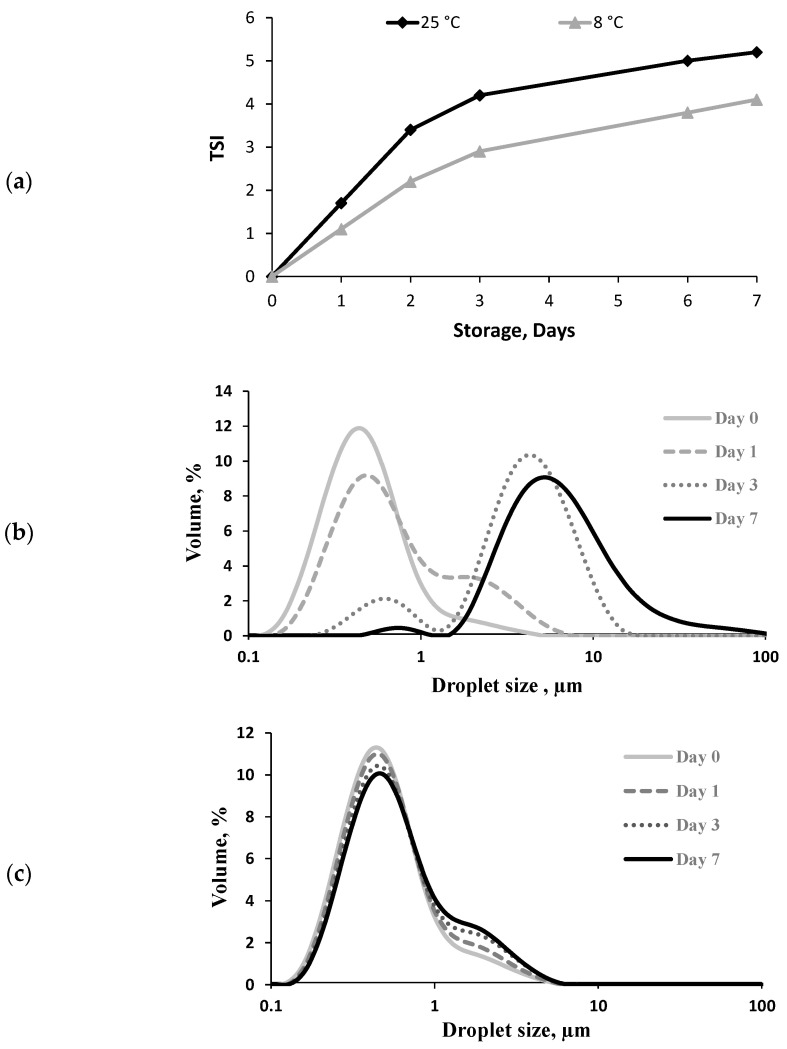
Physical stability of fish oil-in-water emulsion: (**a**) evolution of TSI (Turbiscan Stability Index) during storage at 25 and 8 °C, (**b**) droplet size distribution during 7 days of storage at 25 °C, and (**c**) droplet size distribution during 7 days of storage at 8 °C.

Figure 1a shows the microstructure of the simple O_1_/W emulsion, where the oil droplets (in red) are surrounded by an interfacial film of WPH (in green). Furthermore, it was observed that WPH, which was used in excess, also locates in the aqueous phase of the emulsion (Figure 1a). At the pH of the emulsion (pH 8), which is above the pI of WPH (pI of 4.06 ± 0.05 [21]), the interfacial film of WPH is negatively charged, leading to a highly negative zeta potential of −42.4 ± 0.7 mV. The latter favors electrostatic repulsions between oil droplets, reducing flocculation and coalescence [22]. Nevertheless, emulsions suffered global physical destabilization when stored either at 25 or 8 °C, as indicated by the increase in the Turbiscan stability index (TSI) during storage, although more severe physical destabilization occurred when stored at 25 °C compared to 8 °C (Figure 2a). Figure 2b,c show the oil droplet size distributions for the simple emulsions stored at 25 or 8 °C. After production, the emulsion showed an almost monomodal distribution, with a mean peak centered at 0.4 µm and a small shoulder ranging from 1 to 1.5 µm. It was observed that the 20% fish oil-in-water emulsion severally destabilized when stored at 25 °C (Figure 2b), with a considerable increase in D_4,3_ (from 0.706 ± 0.125 at day 0 to 9.035 ± 0.658 µm at day 7). On the other hand, the emulsion remained physically stable during 7 days of storage at 8 °C (Figure 2c), with only a slight increase in D_4,3_ (from 0.725 ± 0.057 at day 0 to 0.897 ± 0.038 µm at day 7). These findings indicate that the oil droplets in the emulsion stored at 25 °C flocculated and coalesced, increasing their size (Figure 2b). Conversely, although creaming and flocculation occurred in the emulsion when stored at 8 °C (Figure 2a), the size of the oil droplets remained practically the same during storage, indicating minimum coalescence (Figure 2c). A decrease in storage temperature increases viscosity, decreases the collisions between droplets, and reduces the difference in density between the oil and water phases, which all favor the physical stability of emulsions [23]. Interestingly, these results expand the potential of WPH to be used as an emulsifier in oil-in-water emulsions of a higher oil content (20 wt.%) in comparison to a previously reported study where WPH was used to stabilize 5% fish oil-in-water emulsions [16].

#### 3.1.2. Physical Stability of Double O_1_/W/O_2_ Emulsions

**Figure 3:**
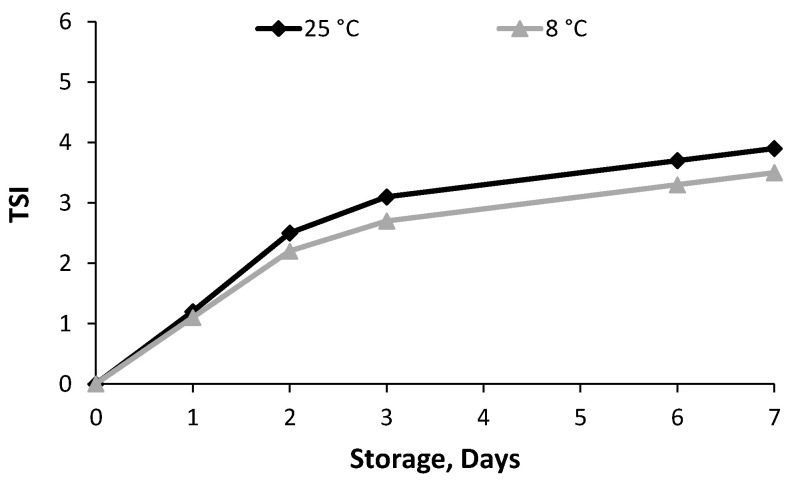
Global physical stability of the fish oil-in-water-in-olive oil double emulsion at two different storage temperature values (8 and 25 °C) given by TSI (Turbiscan Stability Index).

Figure 1b shows the microstructure of the double O_1_/W/O_2_ emulsion. It is observed the dispersion of fish oil droplets within a water phase, which is further dispersed in the outer olive oil phase. The global physical stability of the double emulsions was investigated using the Turbiscan equipment. The results indicate that sedimentation of the droplets of the simple emulsion occurred during storage due to an increase in the backscattering (ABS, %) at the bottom of the vial, which resulted in increasing TSI (Figure 3). Similarly to the simple emulsion, the double O_1_/W/O_2_ emulsion showed slightly higher physical stability at 8 °C when compared to 25 °C. In any case, it should be noted that at both storage temperature values, the TSI value was lower than 4 (Figure 3), denoting that double emulsions were fairly physically stable during 7 days [24]. Alternatively, Diep et al. [25] reported the production of double sunflower oil/water (40–60%)/palm oil emulsions that were physically stable for 4 weeks at 5 °C. These authors used sodium caseinate and k-carrageenan for the stabilization of the emulsions, which were produced by applying fast crystallization after homogenization with UltraTurrax. Interestingly, our results indicate that, even without crystallization of the second oil phase (O_2_), it is feasible to obtain fairly physically stable double emulsions when using whey protein and PGPR as hydrophilic and hydrophobic emulsifiers, respectively. To the best of our knowledge, other previous studies on the production and characterization of double oil-in-water-in-oil emulsions did not report results on the physical stability of the emulsions but only focused on oxidative stability [5,6].

### 3.2. Oxidative Stability of Emulsions

#### 3.2.1. Oxidative Stability of Simple O_1_/W Emulsion

**Figure 4:**
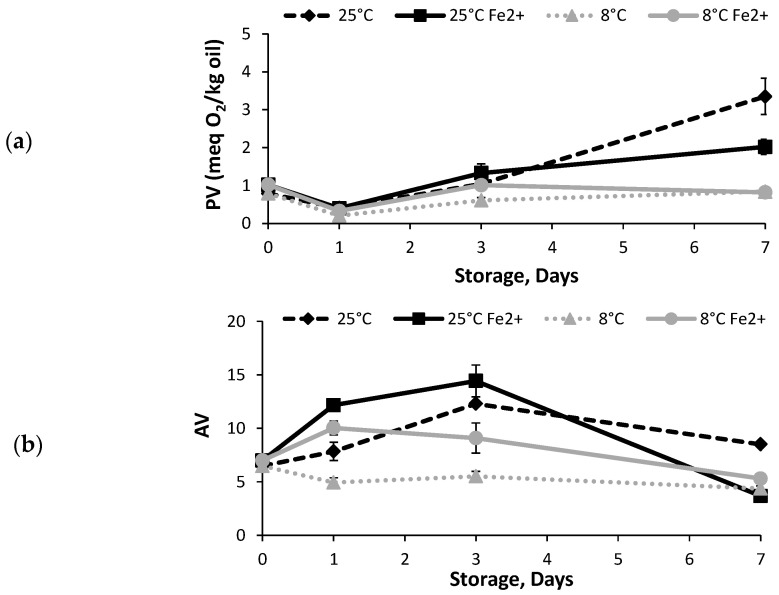
Peroxide value (PV) (**a**) and anisidine value (AV) (**b**) of primary emulsions in a seven day-storage period at 25 °C and 8 °C in the dark.

Figure 4a shows the concentration of primary oxidation products of the simple emulsion during different storage conditions. It was observed that the PV did not considerably increase during production of the emulsion (from 0.47 for fish oil to 1 meq O_2_/kg oil). This denotes that the WPH used had an appropriate oxidative status (lipid content of 2.3 wt.% [16]) since it did not initiate/propagate lipid oxidation of fish oil during emulsion production. The simple emulsion did not show an increase in PV for 7 days when stored at 8 °C, either with addition or not of Fe^2+^ (Figure 4a). When stored at 25 °C, the simple emulsion showed a lag-phase of 3 days either when prooxidants were added or not. After that point, PV slightly increased, reaching a significantly (*p* < 0.05) higher PV (3.3 ± 0.5 meq O_2_/kg oil) for the emulsion without Fe^2+^ when compared to the emulsion with prooxidants (PV of 2.0 ± 0.2 meq O_2_/kg oil). This correlates well with the AV results, with AV measuring secondary oxidation products, such as 2-alkenals and 2,4-alkadienals, which are formed from the decomposition of hydroperoxides [26]. Indeed, emulsions containing Fe^2+^ showed significantly (*p* < 0.05) higher AV (e.g., at day 1) when compared to emulsions without prooxidants independently of the temperature of storage (Figure 4b). The reduction in AV observed (Figure 4b) might be attributed to the potential reaction of these aldehydes with the amino groups of WPH [27,28]. As expected, emulsions stored at 25 °C presented significantly (*p* < 0.05) higher AV when compared to emulsions stored at 8 °C (Figure 4b), which also agrees with the lower physical stability of simple emulsions when stored at 25 °C compared to 8 °C. Physical instability of emulsions denote alterations of the interface microstructure, reducing the ability of this physical barrier to protect the emulsified fish oil [19].

It Is worth mentioning that PV and AV results obtained in this study for the simple emulsion (20 wt.% fish oil) was in the range of those values previously reported for 5 wt.% fish oil-in-water emulsion stabilized with WPH at 20 °C and containing Fe^2+^ [16]. WPH exhibits emulsifying, as well as antioxidant, properties (e.g., chelating and radical scavenging activities) [16,21]. Both antioxidant properties are beneficial for preventing lipid oxidation since radicals can be scavenged at the oil/water interface by the adsorbed WPH-peptides avoiding the propagation of lipid oxidation reactions while metal ions can be chelated in the aqueous phase of the emulsion by the non-adsorbed WPH-peptides [29]. Thus, these results denote the potential of using WPH as an emulsifier for the oxidative stabilization of medium fat (20 wt.%) fish oil-oil-in-water emulsions.

#### 3.2.2. Oxidative Stability of Double O_1_/W/O_2_ Emulsions

**Figure 5:**
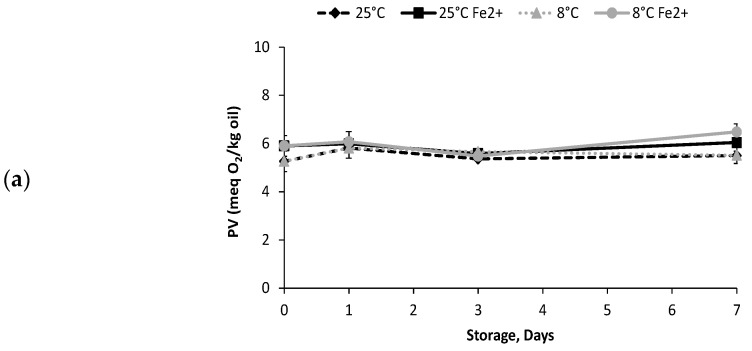

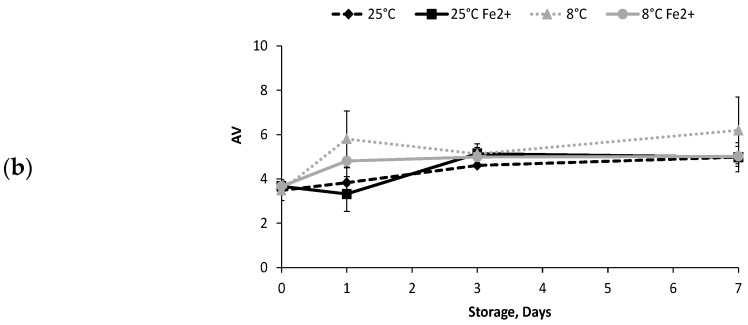
Peroxide value (PV) (**a**) and anisidine value (AV) (**b**) of multiple emulsions in a seven day-storage period at 25 °C and 8 °C in the dark.

Figure 5 shows the PV and AV of the double emulsion at different storage conditions.

Initial PV (meq O_2_/kg) and AV values ranging from 4–6 were observed, which did not significantly (*p* < 0.05) increase during 7 days of storage independently of the temperature of storage and the addition or not of prooxidants. In fact, these initial PV and AV values obtained for the fish oil-in-water-in-olive oil double emulsions (O_1_/W/O_2_) correlate well with the PV and AV of the commercial olive oil used (PV of 5.8 ± 0.2 meq O_2_/kg oil and AV of 6 ± 1). Although olive oil presents natural antioxidants (tocopherols and polar phenolic compounds) [30], storage of the olive oil at supermarket conditions led to its initial oxidation.

Interestingly, these results indicate that the oxidation of the emulsified fish oil did not occur during storage, which denotes the high protecting effect of the WPH used as an emulsifier that also exhibited antioxidant properties at the interface [15,16]. Contrarily, Dwyer et al. [5] reported an increase in PV but not in AV during storage at 5 °C for O_1_/W/O_2_ double emulsions (O_1_: camelina and fish oils, and O_2_: sunflower and palm oils) when using sodium caseinate and PGPR as hydrophilic and hydrophobic emulsifiers, respectively. The authors attributed the increase in the concentration of lipid hydroperoxides in the double emulsion to lipid oxidation occurring in the external oil phase (O_2_), which was heated to 50 °C prior to introduction of the simple emulsion. In a follow-up study, and in order to enhance oxidative stability, the same authors [6] investigated the effect of natural antioxidants (hydrophilic: green tea extract and/or hydrophobic: α-tocopherol) on the formation of lipid hydroperoxides and secondary oxidation products in the double emulsions during storage. They found that the addition of green tea extract (containing polyphenols such as epicatechin, epicatechin gallate, epigallocatechin, and epigallocatechin gallate) enhanced the oxidative stability of the double emulsion, which was not observed for the addition of α-tocopherol. These polyphenols have been reported to interact with proteins (e.g., caseins used as hydrophilic emulsifier) [31], which allows their location at the interface where they can effectively scavenge free radicals to reduce lipid oxidation [32]. The latter highlights the importance of locating antioxidants at the oil/water interface of the simple emulsion for improving the oxidative stability of double O/W/O emulsions. This was achieved in our study by the efficient approach of using whey protein hydrolysate exhibiting both emulsifying and antioxidant activity.

## 4. Conclusions

We studied the physical and oxidative stabilities of fish oil-in-water-in-olive oil double emulsion (O_1_/W/O_2_) stabilized with whey protein hydrolysate and polyglycerol polyricinoleate as hydrophilic and hydrophobic emulsifier, respectively. This double emulsion was investigated as a model system for lipid-based matrices enriched with long-chain omega-3 polyunsaturated fatty acids. The whey protein hydrolysate showed high potential for the physicochemical stabilization of medium fat 20 wt.% fish oil-in-water emulsion due to their high emulsifying and antioxidant properties. In addition, the whey protein hydrolysate also inhibited the lipid oxidation of fish oil when the simple fish oil-in-water emulsion was further dispersed in a second oil phase, such as olive oil. Indeed, the double fish oil-in-water-in-olive oil (O_1_/W/O_2_) double emulsions did not significantly increase its peroxide and *p*-anisidine values during storage for 7 days independently of the temperature and addition or not of prooxidants, which remained constant at the initial values of the olive oil used.

## Data Availability

Not applicable.
